# Comment on DKK1 inhibits canonical Wnt signaling in human papillomavirus-positive penile cancer cells

**DOI:** 10.1016/j.tranon.2021.101326

**Published:** 2021-12-30

**Authors:** Haissa O. Brito, José de Ribamar Rodrigues Calixto, Rui Medeiros, Rui M. Gil da Costa

**Affiliations:** a Post-graduate Programme in Adult Health (PPGSAD), Federal University of Maranhão (UFMA), Avenida dos Portugueses, São Luís, Maranhão 65080-805, Brazil; b Department of Morphology, Federal University of Maranhão (UFMA), São Luís, Maranhão 65080-805, Brazil; c Federal University of Maranhão University Hospital (HUUFMA), Brazil; d School of Medicine, Dom Bosco University (UNDB), São Luís, Maranhão 65075-441, Brazil; e Molecular Oncology and Viral Pathology Group, Research Center of IPO Porto (CI-IPOP) / RISE@CI-IPOP (Health Research Network), Portuguese Oncology Institute of Porto (IPO Porto)/Porto Comprehensive Cancer Center (Porto.CCC), Portugal; f Faculty of Medicine, University of Porto, Porto 4200-319, Portugal; g Biomedicine Research Center (CEBIMED), Faculty of Health Sciences, Fernando Pessoa University, Porto 4249-004, Portugal; h Virology Service, IPO-Porto, Porto 4200-072, Portugal; i Centre for the Research and Technology of Agro-Environmental and Biological Sciences (CITAB), Inov4Agro, University of Trás-os-Montes and Alto Douro (UTAD), Quinta de Prados, Vila Real 5000-801, Portugal; j LEPABE, Laboratory for Process Engineering, Environment, Biotechnology and Energy, Faculty of Engineering, University of Porto, Rua Dr. Roberto Frias, Porto 4200-465, Portugal

Penile squamous cell carcinoma is a neglected disease whose global distribution reflects the health disparities between developed and developing countries. This type of cancer most commonly affects developing countries in Africa and South America with age-standardized incidence rates reaching up to 6.15 per 100,000 in certain areas where penile cancer constitutes a significant public health concern [1]. The current classification of penile cancer broadly distinguishes between two forms of the disease, one induced by human papillomavirus (HPV) infection and another presumably driven by poor hygiene and chronic inflammation as observed, e.g. in patients with untreated phymosis [2]. The first form of the disease is characterized by the presence of HPV DNA, expression of HPV oncogenes, overexpression of p16^INK4a^ and includes specific histological subtypes most frequently showing warty or basaloid morphology and viral cytopathic effects. Conversely, non-HPV related penile cancer shows distinctive histological morphology (e.g. the "usual" histological subtype) and molecular characteristics, typically with no p16^INK4a^ accumulation. Two large scale studies have estimated the worldwide proportion of HPV-related penile cancers at 30–50% of total penile SCCs, but the frequency of HPV-related versus HPV-negative disease varies greatly between different world regions [3,4]. Histological grading and TNM staging are key elements to establish the prognosis of penile cancer patients, While localized lesions may be treated surgically, high-grade lesions and those showing lymph node involvement are eligible for chemoradiotherapy, often involving platinum-based chemotherapy. However, platinum chemoresistance via loss of p53 and phosphatase and tensin homolog (PTEN) or via up-regulation of the epithelial growth factor receptor (EGFR) is common and the prognosis for lymph node-positive patients remains dismal, as recently reviewed [5]. The rarity of the disease and its low incidence in developed countries, together with the paucity of models for pre-clinical research have long delayed the development of new therapeutic approaches for penile cancer patients. However, a number of ongoing clinical trials are now testing new agents or drug combinations in penile cancer patients, as previously reviewed. Multiple *in vitro* and *in vivo* models for translational research have also been reported in recent years [6], although none are available commercially. Among these are the first cell lines representing HPV-positive penile cancer, reported by a German team. In the present issue of *Translational Oncology*, the same research team reports the use of this new research tool to provide important insights into the regulation of signaling via the wingless-type integration site (Wnt) pathway and its biopathological implications in HPV-related penile cancer [7]. this work is a great example of how the newly described preclinical models can contribute for translational research on penile cancer. Previous studies suggested that Wnt signaling was active in penile cancer regardless of its HPV status [8], although its possible association with disease outcome remained obscure. Interestingly, a recently disclosed mouse model of HPV-negative penile cancer partially relies in adenomatous poliposis coli (*Apc*) activating mutations leading to Wnt activation, in cooperation with *Smad4* mutations [9]. Wnt signaling is highly significant for cancer progression and in some solid malignancies Wnt promotes chemorresistance [10], which remains a major challenge in penile cancer as discussed in the previous paragraphs. Therefore, determining the role of Wnt in penile cancer is an important issue with potentially major clinical implications. The authors employed three cell lines representing a primary and two lymph node metastases from patients with HPV-positive penile cancer to study the transcriptional status of the Wnt pathway and observed an up-regulation of markers related to canonical Wnt signaling, compared with normal foreskin keratinocytes. In an elegant experiment employing 3D raft organotypic cell cultures, normal foreskin keratinocytes transduced with the HPV16 E6 and E7 oncogenes showed significantly up-regulated protein expression of the Wnt enhancer leucine rich repeat containing G protein coupled receptor 6 (LGR6). This was associated with higher expression of Wnt target genes such as SRY-box transcription factor 2 (SOX2) and octamer-binding transcription factor 4 (OCT4). These observations support the hypothesis that HPV16 may enhance Wnt signaling via upregulation of LGR proteins. Surprisingly, neither HPV-positive cells nor HPV-positive and HPV-negative penile cancer samples from Russian and German patient cohorts showed nuclear translocation of the pivotal Wnt factor β-catenin. A reporter gene assay also failed to show β-catenin nuclear translocation in response to stimulation with Wnt3. These observations were at odds with the author's previous findings and suggested that, despite the increased expression of Wnt ligands and receptors, there is actually no active Wnt signaling in HPV-positive penile cancer. The authors explained this discrepancy by the accumulation of Wnt inhibitors, particularly Dickkopf 1 (DKK1), which was found at high levels in HPV-positive cell lines and was able to inhibit Wnt signaling *in vitro*, as shown by gene reporter assays. In this context, the authors propose that DKK1 expression is driven by SOX2, itself upregulated by HPV16 E7, promoting cancer cell stemness and immune evasion. Importantly, within HPV-positive clinical specimens, DKK1 accumulation was strongly associated with higher histological grade and TNM stage, supporting its potential role as a biomarker for tumor aggressiveness. Additional studies are required to determine the potential associations of DKK1 expression in blood and tumor tissues with disease outcome.

Overall, this remarkable study by Bley et al. adds new layers of complexity to our understanding of Wnt regulation on penile cancer. The author's approach also highlights the value of newly developed models for translational research on this neglected disease. The use of other complementary models, representing HPV-positive and HPV-negative disease, should be able to further elucidate the role of Wnt regulators in penile cancer. Given the limited availability of penile cancer cell lines, international cooperation efforts are urgently needed to bridge this gap.

## CRediT authorship contribution statement

**Haissa O. Brito:** Writing – original draft, Writing – review & editing. **José de Ribamar Rodrigues Calixto:** Writing – review & editing. **Rui Medeiros:** Writing – review & editing. **Rui M. Gil da Costa:** Conceptualization, Writing – original draft, Writing – review & editing.

## Declaration of Competing Interest

The authors have no conflicts of interest to declare.
